# Impact of of steaming on lipid profile of 10 sweetpotato varieties: LC-ESI-MS/MS comparative analysis

**DOI:** 10.1016/j.fochx.2025.103159

**Published:** 2025-10-14

**Authors:** Xia Jiang, Yanqiang Yao, Rong Zhang, Yujuan Zhong, Zhangying Wang

**Affiliations:** a Crops Research Institute, Guangdong Academy of Agricultural Sciences & Key Laboratory of Crop Genetic Improvement of Guangdong Province, Guangzhou, Guangdong 510640, China; b Vegetable Research Institute, Guangdong Academy of Agricultural Sciences/, Guangdong Key Laboratory for New Technology Research of Vegetables, Guangzhou 510640, China

**Keywords:** LC-ESI-MS/MS, lipid, multivariate analysis, sweetpotato, steaming

## Abstract

Lipids play essential roles in cell membrane composition and metabolic processes in plants. In this study, the lipid metabolites of 10 sweetpotato varieties, both before and after being steamed for 40 min, were investigated for the first time using LC-ESI-MS/MS. 581 lipid metabolites were identified, primarily comprising fatty acids, glycerophospholipids, glycerolipids and sphingolipids. The lipid content across all varieties ranged from 2781 to 5335 μg/g, with varieties G79 and P32 exhibiting consistently higher lipid levels in raw and steamed samples. 354 differentially accumulated lipid metabolites were identified among the varieties, predominantly triglycerides within the glycerolipid class. After steaming, the fatty acids content of all varieties decreased significantly by 38–1004 μg/g, while the glycerolipids content increased significantly. Correlation analysis suggested that lipids may influence the flavor characteristics of sweetpotato. This study provides a comprehensive analysis of sweetpotato lipid metabolism and offers a theoretical foundation for future breeding and quality improvement efforts.

## Highlights

• A total of 354 lipid DAMs were identified among sweetpotato varieties.
• The lipid content of G79 was 1.3 to 1.8 times that of the other sweetpotatoes.
• Fatty acids content decreased and glycerolipids content increased after steaming.
• FAs, TGs, DGs affect the flavor of sweetpotato to a certain extent.

## 1 Introduction

Sweetpotato (Ipomoea batata [L.] Lam.) holds a significant position in global agricultural production and food supply systems as an important food and cash crop worldwide (Zhao et al., 2025). Table-stock sweetpotato is commonly consumed as a staple food in China (Xie et al., 2022). In 2023, China produced 51.6 million tons of sweetpotato, accounting for 55.21 % of global production (https://www.fao.org/faostat/en/). Beyond its role as a staple food, sweetpotato is also valued for its rich nutritional profile, including carbohydrates, dietary fiber, vitamins, minerals, carotenoids, and anthocyanosides. These components exhibit antioxidant, anti-inflammatory, and immune-enhancing properties, and may help reduce the risk of cardiovascular diseases, prevent cancer, and aid in weight management (Qin et al., 2022; Wang, 2024).

Lipids constitute a class of water-insoluble organic molecules, serving as vital structural components of plant biomembranes and fulfilling essential roles in energy storage and transport, signal transduction, and other biological processes (Chen et al., 2024; Zhang et al., 2024). Based on their chemical backbone and biochemical properties, lipids are classified into eight major categories: fatty acids, glycerolipids, glycerophospholipids, sphingolipids, sterol lipids, prenol lipids, saccharolipids, and polyketides (Fahy et al., 2005). In sweetpotato, lipids account for approximately 2.7 % of dry weight, primarily comprising neutral lipids, glycolipids, and phospholipids (Walter et al., 1971). As integral components of cellular membranes, lipids participate in numerous metabolic pathways in sweetpotato, playing crucial roles in substance transport and signaling. Furthermore, the lipid composition of these membranes dynamically responds to environmental changes (de Jong & Munnik, 2021; Seth et al., 2024). Lipids significantly influence the edible quality and processing characteristics of sweetpotato. Specifically, the types and levels of distinct lipid components are closely associated with key edible quality attributes, such as flavor and taste (Zhou et al., 2002; Zhou et al., 2024).

During heat treatment, certain lipids undergo chemical reactions such as oxidation and degradation, yielding compounds that contribute to unique flavors and significantly influence the aroma profile of sweetpotato. Notably, unsaturated fatty acids are precursors to a majority of the volatile organic compounds (VOCs) produced, including aldehydes, alcohols, ketones, and other related molecules (Zhang et al., 2023). Furthermore, the formation of starch-lipid complexes can modulate the textural properties of sweetpotato by altering starch characteristics, thereby impacting its perceived taste (Gu et al., 2011). Therefore, elucidating the lipid composition of sweetpotato and its intricate relationship with flavor and taste is crucial for understanding and ultimately enhancing these key quality attributes.

Earlier studies have commonly employed Soxhlet extraction or the Bligh−Dyer method, among others, to extract crude lipids from sweetpotato. However, these conventional approaches are associated with methodological limitations, such as demanding pretreatment requirements, which can introduce significant deviations in the results (Manirakiza et al., 2001). Lipidomics, a branch of metabolomics, has emerged as a powerful discipline for the comprehensive characterization and quantification of lipid molecules within biological systems (Zhang et al., 2021). A common approach in lipidomics is the qualitative and quantitative detection of lipid molecules in organisms by nuclear magnetic resonance (NMR), gas chromatography–mass spectrometry (GC–MS) and liquid chromatography-mass spectrometry (LC-MS) (Tao et al., 2024). Determination of lipids in plants based on mass spectrometry allows the characterization and tracing of the lipid composition of plants (Sun et al., 2020). Previously, ESI-MS was used to identify the lipids in the leaves and roots of two sweetpotato varieties under salt stress, and 524 and 330 lipid molecules were detected, respectively (Yu et al., 2019). However, it was mainly used to study the lipid composition of sweetpotato leaves under salt stress. At present, Liquid Chromatography-Electrospray Ionization-Mass Spectrometry (LC-ESI-MS/MS) has not been applied to analyze the lipidomics characteristics of sweetpotato tubers. And so far, there have been no reports on the lipidomic composition of sweetpotato tubers. Therefore, it is important to study the lipid profile of sweetpotato and the changes in sweetpotato lipids after steaming.

Currently, we used LC-ESI-MS/MS to characterize and quantify lipids in the tubers of 10 sweetpotato species before and after steaming. The aim of the present study was to reveal the lipid composition of sweetpotato tubers and the effect of steaming on sweetpotato lipids, and to analyze the association between lipids and key volatile organic compound of sweetpotato by correlation heat map. This study fills the gap of lipidomics research on sweetpotato tubers and processing, and provides a theoretical basis for improving sweetpotato quality characteristics and optimizing sweetpotato breeding.

## 2 Materials and methods

### 2.1 Chemicals and reagents

HPLC-grade acetonitrile (ACN), methanol (MeOH), isopropanol (IPA), dichloromethane, and methyl tert-butyl ether (MTBE) were purchased from Merck (Darmstadt, Germany). HPLC-grade formic acid (FA), Ammonium formate(AmFA) were purchased from Sigma-Aldrich (St. Louis, MO. USA). Milli-Q Water Purification System (Millipore, MA, USA) was used to make ultrapure water. Lipid standards were purchased from Sigma-Aldrich (St. Louis, MO. USA) or Avanti Polar Lipids (Alabaster, AL).

### 2.2 Plant material, and sample preparation

The Jieshu 95–16 (J95–16), Guangshu 79 (G79), Pushu 32 (P32), Anna (AN), Guangshu 87 (G87), Guangshu 20 (G20), Guangzishu 1 (GZ1), Guangzishu 10 (GZ10), Zhanzishu 3 (ZZ3), Guangzishu 2 (GZ2) were supplied by the national germplasm of Guangzhou Sweetpotato Nursery, and planted with standard agricultural practices in the experimental field of Zhanjiang Academy of Agricultural Sciences in Zhanjiang, China. J95–16, AN, G87 are yellow-fleshed sweetpotatoes, G79, P32 are orange-fleshed sweetpotatoes, and G20, GZ1, GZ10, ZZ3, GZ2 are purple-fleshed sweetpotatoes. Root samples were harvested 150 days after planting.

Six distinct varieties of medium-sized sweetpotatoes were selected, thoroughly washed with distilled water, dried, and then weighed. Following the washing and drying process, the sweetpotatoes were randomly divided into two experimental groups: one designated for raw sample analysis and the other for steamed sample analysis. Each group consisted of three biological replicates to ensure data reliability and reproducibility. The steamed sample was obtained by steaming the sweetpotatoes for 40 min. All samples were freeze-dried for 72 h and then ground into powder. The dried samples were sieved through an 80-mesh sieve and stored in a sealed bag at −20 °C.

### 2.3 Lipids extraction

Lipids were extracted using a methanol, methyl tert-butyl ether (MTBE) solvent system (Liu, Guo, et al., 2023). 20 mg dry sample was added to a 2 mL centrifuge tube, put in a steel ball, and 1 mL lipid extract (MTBE:MeOH = 3:1) was added, vortexed for 30 min. Then, 300 μL ultrapure water was added, vortexed for 1 min and left the sample at 4 °C for 10 min. After centrifugation at 12000 r/min for 3 min at 4 °C, 400 μL supernatant was transferred to a 1.5 mL centrifuge tube and concentrated at 20 °C until completely dry. 200 μL lipid complex solution (ACN:IPA = 1:1) was added to redissolve, vortexed for 3 min, and centrifuged at 12000 r/min at 4 °C for 3 min. Finally, 120 μL reconstituted solution was collected for LC-MS/MS analysis.

### 2.4 Quality control

The mixed solution of all samples was extracted in equal volume as a quality control (QC) sample. In the process of instrumental analysis, a QC sample is detected every 10 samples to determine the stability of the instrument during the detection period.

### 2.5 HPLC conditions

The sample extracts were analyzed using an LC-ESI-MS/MS system (UPLC, ExionLC™ AD, MS, QTRAP®6500+ System). LC separation was performed on an Accucore™ C30 column (2.6 μm, 2.1 mm × 100 mm i.d.) using a binary solvent system consisting of phase A (ACN:water = 60/40, *V*/V, 0.1 %FA, 10 mmol/L AmFA) and phase B (ACN:IPA = 10/90, V/V, 0.1 %FA, 10 mmol/L AmFA). The gradient elution program was as follows: 0 min, 80 %A; 2 min, 70 %A; 4 min, 40 %A; 9 min, 15 %A; 14 min, 10 %A; 15.5 min, 5 %A; 17.3 min, 5 %A; 17.5 min, 80 %A; 20 min, 80 %A; flow rate, 0.35 mL/min; temperature, 45 °C; injection volume: 2 μL.

### 2.6 ESI-MS/MS conditions

Mass spectrometry analysis was performed on a triple quadrupole-linear ion trap mass spectrometer (QTRAP) in the positive and negative ionization modes. The electrospray ionization (ESI) conditions were set as follows: ion source, turbo spray; source temperature 500 °C; ion spray voltage (IS) 5500 V (Positive), −4500 V(Neagtive); Ion source gas 1 (GS1), gas 2 (GS2), curtain gas (CUR) were set at 45, 55, and 35 psi, respectively. Instrument tuning and mass calibration were performed with 10 and 100 μmol/L polypropylene glycol solutions in the triple quadrupole (QQQ) and linear ion trap (LIT) modes, respectively. QQQ scans were acquired as multiple reaction monitoring (MRM) experiments with collision gas (nitrogen) set to 5 psi. Declustering potential (DP) and collision energy (CE) for individual MRM transitions was done with further DP and CE optimization. A specific set of MRM transitions were monitored for each period according to the metabolites eluted within this period.

### 2.7 Quantification analysis

Based on the self-built metware database, the information was qualitatively analyzed according to the retention time (RT), the parent ion and the daughter ion of the detected substance. Lipid quantification was achieved by MRM analysis using a QQQ mass spectrometry. After obtaining the lipid mass spectrometry analysis data of the sample, the peak area of all the substance peaks was integrated, and the quantitative analysis was carried out by the internal standard method.

### 2.8 Statistical analysis and visualization

SPSS22.0 (SPSS-IBM Chicago, IL, USA) was used for One-way analysis of variance (ANOVA) with a significance level of 0.05. The heatmap was visualized by TBtools (Toolbox for Biologists; version 1.113, China), correlation heat maps were analyzed by Origin 2022 (OriginLab Corporation, MA, USA) and unsupervised principal component analysis (PCA) plots, differential metabolite screening and OPLS-DA score map and correlation network map was performed using the Metware Cloud (https://cloud.metware.cn). All analysis were repeated in triplicate to ensure accuracy and all mapping data are standardized.

## 3 Results and discussion

### 3.1 Reliability of the analytical method

**Fig. 1:**
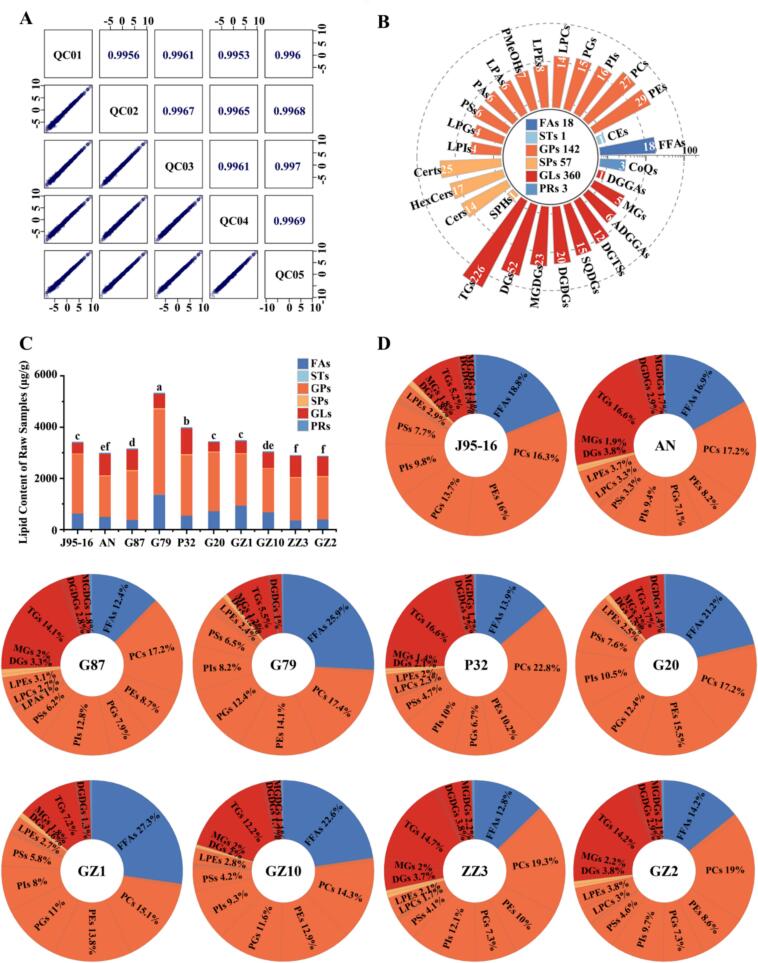
Reliability of lipid assays and overview of lipid subclasses and categories for 10 sweetpotato species. (A) Correlation of QC samples. (B) Number of lipids identified in 29 lipid subclasses and 6 lipid subcategories. (C) Category stacking plot of total lipid content of 10 sweetpotato species. (D) Percentage of subclass lipid content of 10 sweetpotato species. Statistically significant differences based on *t*-test. Different letters of a, b, c, d, e, f and g indicate that there were significant differences in the results.

**Supplementary Fig S1:**
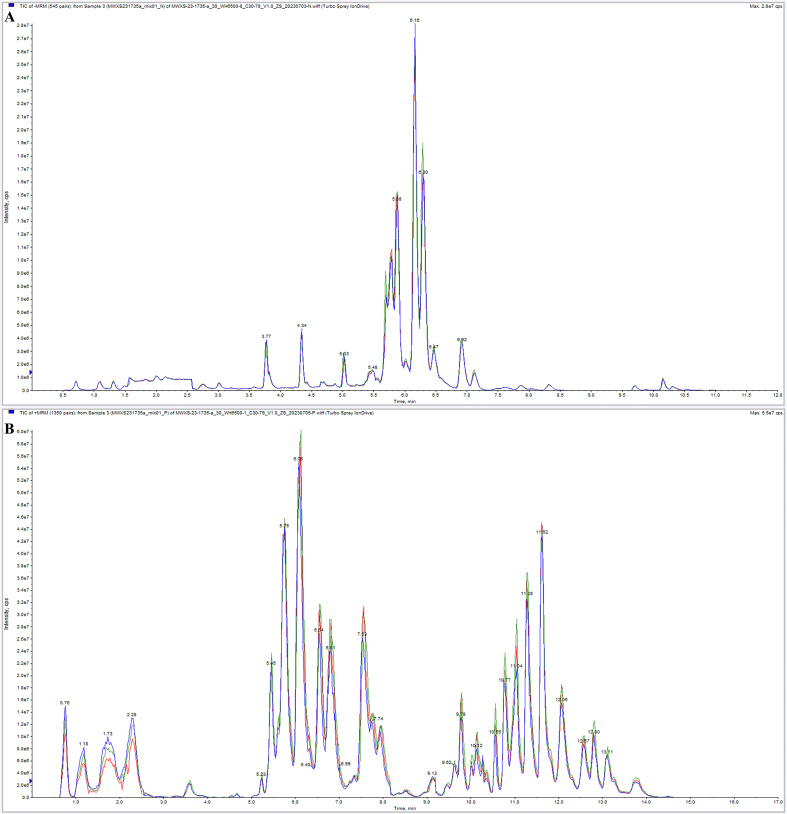

The lipid mass spectra of 10 sweetpotato varieties were detected based on UPLC-ESI-MS/MS. For the reliability of the data, the total ion current (TIC) diagram of the same QC sample was analyzed by overlapping display analysis (Fig.S1). The Fig.S1 showed that the TIC curve had high overlap, and RT and peak intensity was consistent, indicating that the instrument had high stability and the experimental data had high reliability. The Pearson correlation analysis of QC samples was shown in Fig. 1A. The correlation coefficients were all above 0.995, indicating the high quality of the data in this study.

### 3.2 Lipidomics profiles of 10 raw sweetpotato samples

In order to have a comprehensive understanding of the composition of lipids in sweetpotato, we determined lipids in 10 raw sweetpotato samples. A total of 581 lipids were detected in 10 sweetpotato varieties, which were categorized into six classes: glycerolipids (GLs, 360), glycerophospholipids (GPs, 142), sphingolipids (SPs, 57), fatty acids (FAs, 18), isoprenol lipids (PRs, 3), and sterol lipids (STs, 1) (Table S1, Fig. 1B). These 581 lipids were subdivided into 28 subclasses, of which GLs mainly included 226 triglycerides (TGs), 52 diglycerides (DGs), 23 monosaccharide diglycerides (MGDGs), 20 disaccharide diglycerides (DGDGs), and 5 monoglycerides (MGs). The main ones in GPs were 29 phosphatidylethanolamines (PEs), 27 phosphatidylcholines (PCs), 14 lysophosphatidylcholines (LPCs), 8 lysophosphatidylethanolamines (LPEs), 7 phosphatidyl methanols (PMeOHs), 6 lysophosphatidic acids (LPAs), 6 phosphatidic acids (PAs). There were only four subclasses in the SPs, which were 25 phytoceramides (Certs), 17 hexose ceramides (HexCers), 14 ceramides (Cers) and 1 sphingol (SPH). FAs, PRs, and STs all had only one subclass, 18 free fatty acids (FFAs), 3 coenzyme Q (CoQ), and 1 cholesteryl ester (CE), respectively (Fig. 1B).

In this study, lipid contents in 10 raw sweetpotato samples were quantified using the internal standard method through peak area integration. The results showed that the lipid contents of the 10 raw sweetpotato samples ranged from 2877 to 5332 μg/g (Fig. 1C, Table 1), accounting for 0.3–0.5 % (dry weight) of the sweetpotato samples. Among them, G79 had the highest lipid content (5331.69 μg/g), which was about 1.3–1.8 times higher than that of the rest of sweetpotatoes (2877.59–3988.84 μg/g). FAs and GPs were the main components of sweetpotato lipids, which constitute about 70 %–88 % of sweetpotato lipids. FAs is mainly synthesized in plastids and can degrade to produce some volatile organic compounds, while GPs is the most common phospholipid, which is amphiphilic and the main component of cell membranes (Seth et al., 2024; Shahidi & Hossain, 2022). The FAs content of G79 (1379.55 μg/g) was significantly higher than that of the other sweetpotatoes, followed by GZ1 (950.27 μg/g). However, the FAs proportion in GZ1 (27.27 %) exceeded that in G79 (25.87 %). The FAs content of G87, ZZ3 and GZ2 (370–409 μg/g) was significantly lower than that of other sweetpotatoes, and their FAs proportion was only around 13 %. The GPs content of G79 (3329.93 μg/g) was also significantly higher than that of other sweetpotatoes (1594–2370 μg/g). Notably, J95–16 (2318.95 μg/g) and G20 (2302.69 μg/g) displayed the highest GP proportions, at approximately 67 %. GL is formed by esterification of glycerol skeleton and FAs (Michaud et al., 2017). The content of GLs in raw sweetpotatoes ranged from 356 to 1002 μg/g, and its proportion ranged from 10 to 28 %, with P32 (1001.88 μg/g) having the highest content, AN, G87, ZZ3, and GZ2 differing insignificantly, and G20 (356.27 μg/g) and J95–16 (408.00 μg/g) having the lowest content. SPs accounted for a relatively low proportion (1 %) of sweetpotato lipids, with levels ranging from 20 to 47 μg/g, while PRs and STs had lowest levels (0.38–20 μg/g), with proportion of only about 0.4 % and 0.02 %, respectively. Plant defense-related proteins are mainly clustered in structural domains consisting of SPs and ST (Mongrand et al., 2010).

**Table 1:** The classes of lipids in raw and steamed sweetpotatoes^a^.

	FA	GP	SP	GL	PR	ST	Total lipid
R-J95–16	642.26 ± 45.68 d	2318.95 ± 89.95 b	33.98 ± 4.11 c	408.00 ± 25.18 g	15.62 ± 0.15 e	1.05 ± 0.03 b	3419.85 ± 165.10 c
R-AN	505.56 ± 45.01 e	1594.38 ± 103.68 d	36.53 ± 2.73 c	837.14 ± 50.87 b	14.33 ± 0.50 f	0.46 ± 0.04 de	2988.39 ± 202.84 ef
R-G87	390.13 ± 25.69 f	1904.83 ± 186.50 c	47.64 ± 2.46 a	795.73 ± 62.72 bc	17.40 ± 0.98 c	0.47 ± 0.03 de	3156.20 ± 278.37 d
R-G79	1379.55 ± 45.22 a	3329.93 ± 156.63 a	47.40 ± 6.63 a	553.56 ± 30.20 e	20.05 ± 0.80 a	1.20 ± 0.03 a	5331.69 ± 239.51 a
R-P32	552.68 ± 13.44 e	2370.78 ± 179.34 b	44.55 ± 2.56 ab	1001.88 ± 49.07 a	18.38 ± 0.05 b	0.56 ± 0.05 d	3988.84 ± 244.50 b
R-G20	730.68 ± 5.42 c	2302.69 ± 94.14 b	35.31 ± 3.69 c	356.27 ± 18.79 h	13.99 ± 0.59 f	0.82 ± 0.06 c	3439.77 ± 122.69 c
R-GZ1	950.27 ± 35.27 b	2014.83 ± 75.19 c	34.08 ± 4.43 c	468.74 ± 29.33 f	15.83 ± 0.77 de	1.04 ± 0.17 b	3484.78 ± 145.16 c
R-GZ10	689.98 ± 6.01 cd	1717.18 ± 82.12 d	20.84 ± 1.31 d	603.00 ± 54.80 d	14.30 ± 0.10 f	1.01 ± 0.04 b	3046.31 ± 144.37 de
R-ZZ3	370.32 ± 32.65 f	1666.10 ± 63.48 d	40.48 ± 4.71 bc	808.95 ± 56.70 b	16.63 ± 0.11 cd	0.44 ± 0.04 de	2902.91 ± 157.68 f
R-GZ2	408.55 ± 77.90 f	1661.19 ± 70.52 d	36.12 ± 2.19 c	756.76 ± 113.94 c	14.60 ± 0.21 f	0.38 ± 0.04 e	2877.59 ± 264.80 f
S-J95–16	456.45 ± 58.82 a	1996.18 ± 106.54 d	22.85 ± 2.00 ef	490.16 ± 66.23 g	12.20 ± 0.65 de	0.92 ± 0.09 a	2978.75 ± 234.32 d
S-AN	415.73 ± 36.20 abc	1817.55 ± 105.25 e	33.95 ± 1.59 c	854.99 ± 40.60 cd	8.33 ± 0.40 f	0.48 ± 0.08 b	3131.03 ± 184.12 c
S-G87	348.71 ± 13.97 bcd	1980.53 ± 85.06 d	43.46 ± 2.71 b	917.86 ± 60.63 b	10.99 ± 0.30 e	0.40 ± 0.04 b	3301.95 ± 162.71 b
S-G79	375.09 ± 3.71 abcd	3149.36 ± 83.75 a	35.34 ± 2.01 c	830.24 ± 32.12 d	14.83 ± 0.61 b	1.07 ± 0.29 a	4405.93 ± 122.48 a
S-P32	426.97 ± 60.48 ab	2507.11 ± 81.22 b	51.80 ± 2.59 a	1311.69 ± 68.13 a	16.23 ± 0.72 a	0.52 ± 0.04 b	4314.31 ± 213.18 a
S-G20	365.95 ± 48.26 bcd	2234.77 ± 135.85 c	28.66 ± 4.97 d	517.80 ± 45.99 g	13.15 ± 0.69 cd	1.00 ± 0.29 a	3161.32 ± 236.05 c
S-GZ1	367.13 ± 54.08 bcd	2223.34 ± 201.13 c	26.87 ± 3.95 de	627.47 ± 55.40 f	12.74 ± 0.99 cd	0.86 ± 0.16 a	3258.41 ± 315.71 bc
S-GZ10	369.71 ± 30.91 bcd	2005.96 ± 91.52 d	19.72 ± 3.23 f	741.21 ± 28.53 e	13.13 ± 0.94 cd	1.03 ± 0.23 a	3150.77 ± 155.36 c
S-ZZ3	331.59 ± 33.27 cd	1940.65 ± 140.56 d	47.90 ± 2.94 ab	835.08 ± 69.02 d	13.71 ± 1.07 bc	0.44 ± 0.12 b	3169.37 ± 246.98 c
S-GZ2	301.34 ± 57.74 d	1553.40 ± 67.45 f	30.76 ± 0.95 cd	892.92 ± 76.47 bc	9.13 ± 0.29 f	0.39 ± 0.08 b	2787.93 ± 202.98 e

a: Different letters of a, b, c, d, e, f, g and h indicate that there were significant differences in the results. The variance analysis in the raw samples and the steamed samples were conducted independently.

Analysis of lipid subclasses (Fig. 1D, Table S1) revealed that FFAs constituted the sole subclass within the FAs category. Although FFAs comprised only 18 individual compounds, they accounted for 12 % to 27 % of the total sweetpotato lipids and represented the predominant subclass in varieties J95–16, G79, G20, GZ1, and GZ10. Among them, there were 10 saturated fatty acids and 8 unsaturated fatty acids, and unsaturated fatty acids had a high nutritional value for humans (Liu et al., 2013). The unsaturated fatty acid content of G79, G20 and GZ1 was higher than the saturated fatty acid content, especially in G79, where the unsaturated fatty acid content was 3–10 times higher than that of the rest of the sweetpotatoes except for G20 and GZ1. The content of linoleic acid in G79 was 2–12 times higher than that of other sweetpotatoes, and linoleic acid can be degraded to produce hexanal. Our earlier study also confirmed that the content of hexanal in raw samples of G79 was significantly higher than that of the rest of the sweetpotatoes (Jiang et al., 2024; Li et al., 2012; Yao et al., 2024). And the main subclasses in GPs were PCs, PEs, PGs, and PIs, where PCs and PEs, which are structural membrane phospholipids, can generate PAs under stress conditions via phospholipase-D (Seth et al., 2024). Whereas DGs, MGs, and TGs were predominant in GLs, TGs, while comprising the highest number of individual substances, exhibited low content, accounting for only 3–16 % of sweetpotato lipids. However, they served as the primary reserve of FAs for carbohydrate and energy production during sweetpotato growth (Xu & Shanklin, 2016).

### 3.3 Multivariate analysis of lipidomics data of 10 raw sweetpotato samples

**Fig. 2:**
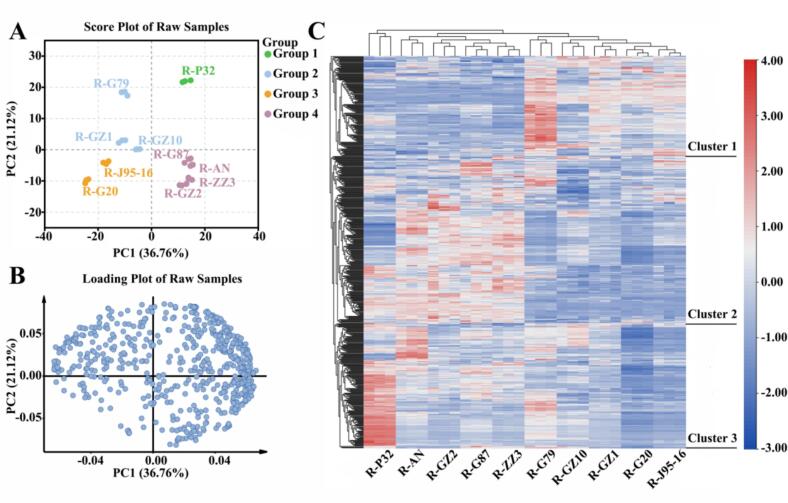
Analysis of lipid metabolites in 10 sweetpotato raw samples. (A) PCA score plot of raw samples, each point represents an independent experimental repeat. (B) PCA load diagram. (C) Cluster heat map visualization of lipid metabolites. The red block represents the up-regulated metabolites, the blue block represents the down-regulated metabolites, and the white block represents the average relative expression intensity of all volatile compounds. (For interpretation of the references to colour in this figure legend, the reader is referred to the web version of this article.)

In order to study the differences in lipid metabolomics of the 10 sweetpotato varieties, we further performed multivariate statistica l analysis of lipidomics data, and the overall principal component analysis (PCA) of 10 sweetpotato varieties was shown in Fig. 2A-B. Good repeatability within each sample group indicates that the results have high reliability and reproducibility. The sum of PC1 (36.76 %) and PC2 (21.12 %) was 57.88 %. Preliminary PCA analysis showed that P32 was well separated from the rest of sweetpotatoes and located in the first quadrant of PCA, while G79, GZ1 and GZ10 were located in the second quadrant of PCA, J95–16 and G20 were located in the third quadrant of PCA, and G87, AN, ZZ3 and GZ2 were located in the fourth quadrant of PCA. The principal component analysis showed that there were some differences in the lipids of the 10 sweetpotato varieties, especially P32 was quite different from the rest of the sweetpotatoes, but the analysis of the PCA results showed that the lipids were not much associated with the flesh-colour of sweetpotato tubers.

Subsequently, a cluster heat map was constructed to observe the relative abundance of lipid metabolites (Fig. 2C). The clustering heatmap results were consistent with the PCA analysis. P32 was distinctly different from the other sweetpotato cultivars. Specifically, AN, GZ2, G87, and ZZ3 formed one cluster, while G79, GZ1, GZ10, G20, and J95–16 formed another cluster. The lipid metabolite accumulation pattern was mainly categorized into three clusters, with lipid metabolites from cluster 1 accumulating more mainly in G79, whereas lipid metabolites from cluster 2 accumulated more in AN, GZ2, G87 and ZZ3. Lipid metabolites of cluster 3 significantly mainly in P32. The differences in lipid metabolite accumulation were able to better distinguish different sweetpotato.

### 3.4 Lipid differentially accumulated metabolites among different sweetpotato raw samples

**Supplementary Fig S2:**
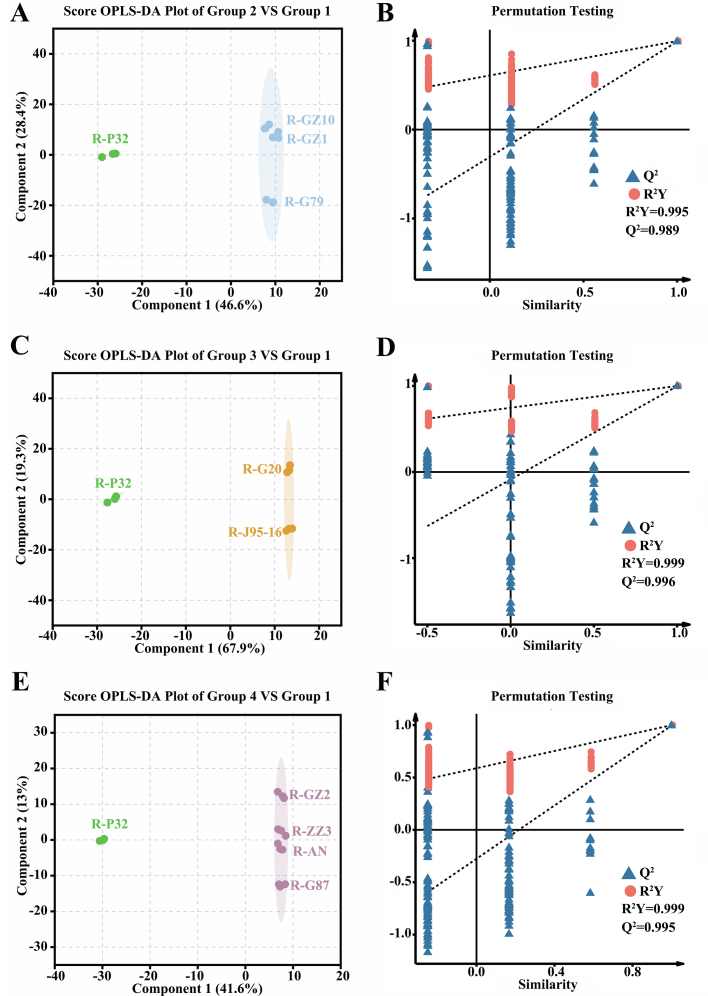

Since PCA can only capture overall differences in lipid metabolites among sweetpotato samples, we divided the 10 samples into four clusters based on the PCA results and clustering heatmap: Group 1 (P32), Group 2 (G79, GZ1, GZ10), Group 3 (J95–16, G20), and Group 4 (AN, G87, ZZ3, GZ2). Using P32 as the reference group, OPLS-DA models were applied to explore the relationships among lipid metabolites across different sweetpotato varieties. Scoring plots (Fig. S2A—F) were then generated to further examine the specific differences in lipid metabolite profiles between these varieties. In the three models, Q^2^ was >0.99, indicating that the constructed models were suitable and has high credibility and good prediction performance. In the OPLS-DA model score plot, there was a clear separation between group 1 (P32) and the rest of sweetpotatoes, indicating that there were some differences in lipid metabolites between group 1 (P32) and the rest of sweetpotatoes. OPLS-DA achieves dimensionality reduction under grouping conditions, and lipid differentially accumulated metabolites (DAMs) can be screened according to the variable influence on projection (VIP) in the model. Lipid DAMs were screened according to the combination of VIP value (≥1), *P* < 0.05 and |log2(fold change)| ≥ 1.

**Fig. 3:**
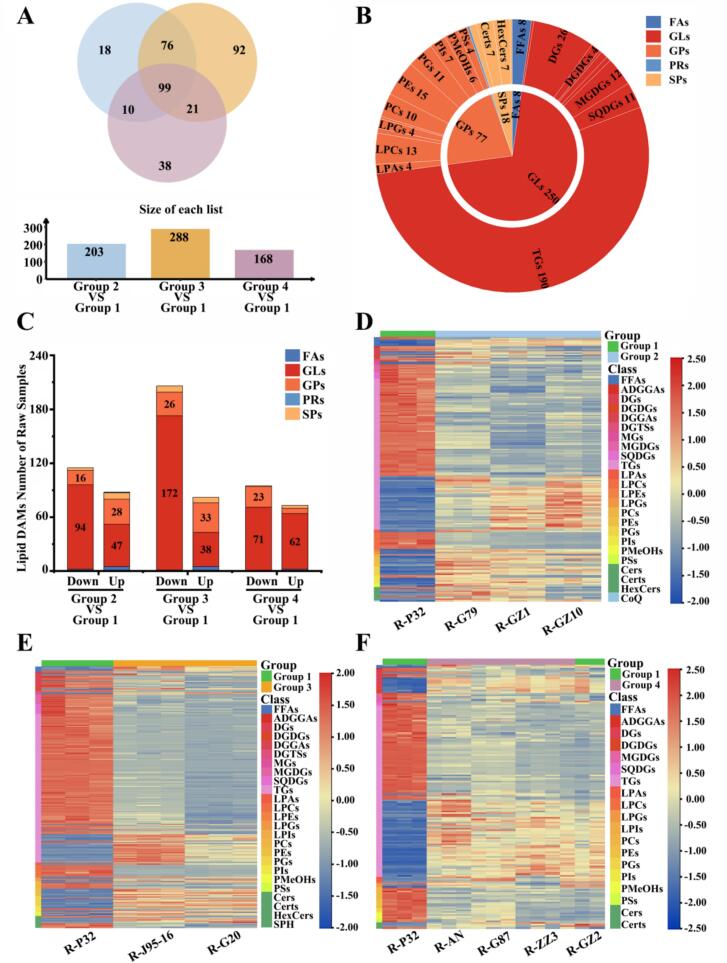
Analysis of lipid differential metabolites in 10 sweetpotato raw samples. (A) Venn diagram showing the distribution of lipid differential metabolites. (B) Lipid differential metabolites category statistics. (C) Statistics of the number of up-regulated and down-regulated subclasses of lipid differential metabolites in group 2, group 3 and group 4 vs group 1. (D—F) Heat map visualization of lipid differential metabolites in group 2, group 3 and group 4 vs group 1. The red square represents the up-regulated metabolites, the blue square represents the down-regulated metabolites, and the yellow square represents the average relative expression intensity of all metabolites.

A total of 354 lipid DAMs were identified in the three comparison combinations, of which 203, 288,168 lipid DAMs were identified in Group 2 vs Group 1, Group 3 vs Group 1, and Group 4 vs Group 1, respectively (Fig. 3A, Table S2–4). A total of 99 common lipid DAMs were identified across the three pairwise comparisons. Specifically, 18, 92, and 31 unique lipid DAMs were found in Group 2 vs Group 1, Group 3 vs Group 1, and Group 4 vs Group 1, respectively. All lipid DAMs were categorized into 5 classes and 26 subclasses based on their chemical structures. Most lipid DAMs belong to GLs and GPs and a small number of lipid DAMs belonged to SPs and FAs, while in the subclasses mainly TGs, DGs, PEs and LPCs had more lipid DAMs (Fig. 3B). The number of lipid DAMs down-regulated was greater than up-regulated in all three pairwise comparisons (Fig. 3C). There were 115, 206, 95 lipid DAMs down-regulated and 88, 82, 73 lipid DAMs up-regulated in group 2 vs group 1, group 3 vs group 1, and group 4 vs group 1, respectively. The number of GLs down-regulated was greater than up-regulated in all three pairwise comparisons, with the highest number of GLs down-regulated in group 3 vs group 1, which was about three times the number of up-regulated ones. In groups 2 vs 1 and group 3 vs group 1, the number of GPs up-regulated was greater than down-regulated, but in group 4 vs group 1, the number of GPs down-regulated was greater than up-regulated.

To visualize lipid differences among sweetpotato cultivars, we generated heatmaps of the lipid DAMs identified in the three pairwise comparisons (Fig. 3D-F). In FFAs, saturated fatty acids were up-regulated in all three comparative combinations. In contrast, unsaturated fatty acids differed only in groups 2 vs group 1 and groups 3 vs group 1, where eicosapentaenoic acid was down-regulated in all of them. There were mainly more TGs DAMs in the three comparison combinations, and the number of TGs down-regulated was greater than that of up-regulated, TGs assumed an important role in the plant's response to environmental factors, which suggests that P32 may be stronger than the rest of the sweetpotatoes in terms of stress tolerance (Yu et al., 2019).

### 3.5 Effect of steaming on the lipids of sweetpotato

**Fig. 4:**
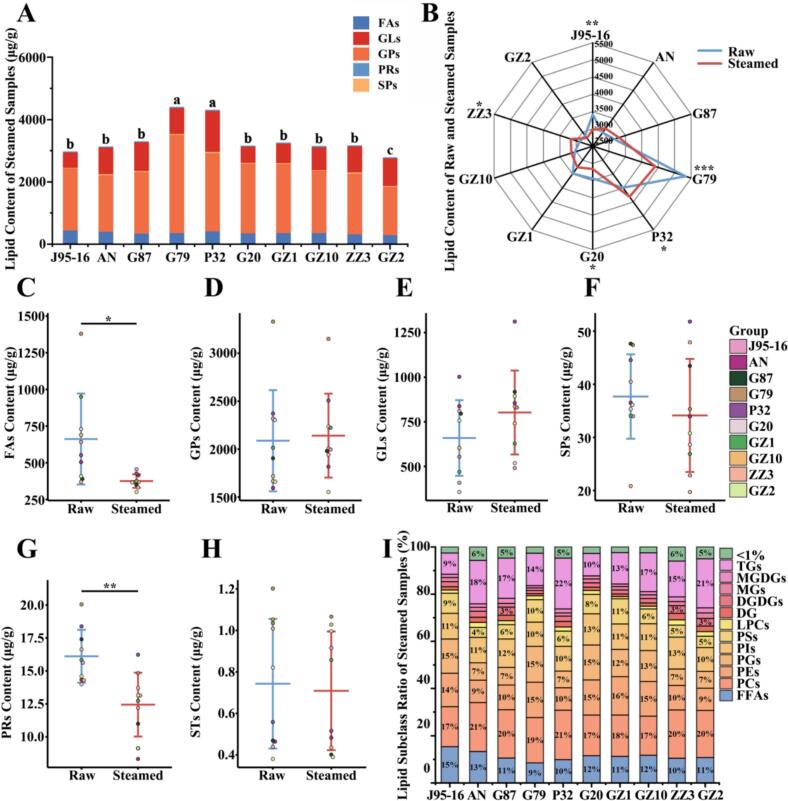
Effect of steaming on sweetpotato lipid metabolites. (A) Category stacking plots of total lipid content of 10 sweetpotato steamed samples. (B) Radar plots of lipid content of sweetpotato before and after steaming. *, ** indicates significant difference in lipid content before and after steaming. (C—H) Box line plots of lipid major categories before and after steaming of sweetpotato. (I) Percentage of subclass lipid content of 10 sweetpotato steamed samples. Statistically significant differences based on *t*-test. Different letters of a, b, c, d, e, f and g indicate that there were significant differences in the results.

Sweetpotato is usually consumed after steaming, and changes in the lipids in sweetpotato after steaming may affect the taste and aroma of sweetpotato. During thermal processing, many changes in sweetpotato quality occur, such as starch pasting and the production of volatile organic compounds (VOCs), which can be related to changes in starch, lipids, etc., in sweetpotato and their interactions (Lian et al., 2022). We determined the lipid metabolites of 10 sweetpotato after steaming, and the results were shown in Fig. 4A and Table 1. The lipid contents of the 10 sweetpotato steaming samples ranged from 2781 to 4405 μg/g, with G79 (4405.93 μg/g) and P32 (4314.31 μg/g) significantly higher than the rest of the sweetpotatoes, and GZ2 (2787.93 μg/g) having the lowest lipid content. The total lipid content of the remaining sweetpotato cultivars showed no significant difference. Following steaming, the total lipid content of G79, J95–16, and G20 decreased significantly, while that of ZZ3 and P32 increased significantly. Notably, G79 exhibited the most substantial reduction, decreasing by 17.36 % relative to its raw sample content (Fig. 4B). FAs and GPs remained the main components of sweetpotato lipids after steaming, but their proportion decreased (66–82 %). The FAs contents after steaming ranged from 301 to 456 μg/g, and the differences in FAs contents among different sweetpotato varieties were not significant. Compared to raw samples, FAs content in steamed samples decreased significantly. Specifically, G79, GZ1, G20, and GZ10 exhibited reductions of 72.81 %, 61.37 %, 49.92 %, and 46.42 %, respectively, relative to their raw samples (Fig. 4C). This decline was likely attributable to FA oxidation during thermal processing, which generates VOCs (Lina & Min, 2022). After steaming, the GPs content of G79 (3149.36 μg/g) was still significantly higher than that of the other sweetpotatoes, being 1.4–2 times that of the other sweetpotatoes. Among them, the one with the lowest GPs content was GZ2 (1553.40 μg/g). Except for J95–16, G79 and GZ2 whose GPs decreased after steaming, the GPs of the other sweetpotatoes increased after steaming, but the difference in GPs between raw and steamed samples was not significant (Fig. 4D). The GLs content of the 10 sweetpotato varieties after steaming was 490–1311 μg/g, which was increased compared with the raw samples (Fig. 4E). Among them, the GLs content of G79, G20, GZ1 and P32 increased by 30–50 % of their own. SPs and STs showed no significant changes after steaming (Fig. 4F, Fig. 4H). PRs decreased significantly after steaming, but due to its low content, it only decreased by almost 1-7 μg/g (Fig. 4G). FFAs, the only subclass of FAs, accounted for 9–15 % of the total lipid content of steamed sweetpotatoes (Fig. 4I). PCs (505–903 μg/g) and PEs (261–661 μg/g) were the subclasses with higher content in GPs, which accounted for about 30 % of the total lipid content of steamed samples. Compared with the raw samples, PCs and PEs increased in steamed samples of most sweetpotato varieties, possibly due to interconversion of some phospholipids during heat treatment (Zhou et al., 2025). Among the GLs, TGs content was the highest (266–929 μg/g) and accounted for 9–22 % of sweetpotato lipids after steaming. There was a significant increase in TGs after steaming, possibly due to the fact that heat treatment causes the phospholipid monolayer encapsulating the lipoproteins to rupture thereby releasing more TGs (Li et al., 2024).

**Fig. 5:**
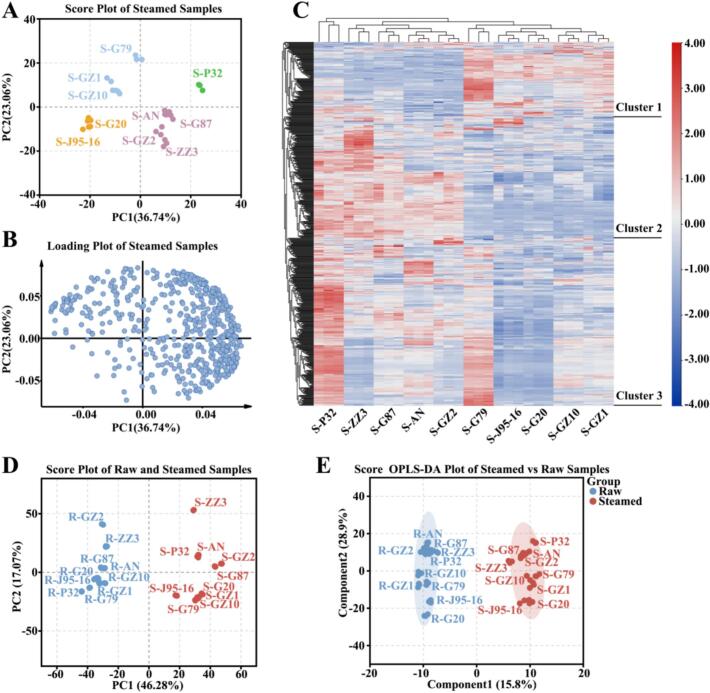
Multidimensional analysis of the effect of steaming on sweetpotato lipids. (A) PCA score plot of steamed samples, each point represents an independent experimental repeat. (B) PCA load diagram. (C) Cluster heat map visualization of lipid metabolites. The red block represents the up-regulated metabolites, the blue block represents the down-regulated metabolites, and the white block represents the average relative expression intensity of all volatile compounds. (D) PCA score plot of raw and steamed samples, each point represents an independent experimental repeat. (F) Score OPLS-DA plot of raw and steamed samples,each point represents an independent experimental repeat. (For interpretation of the references to colour in this figure legend, the reader is referred to the web version of this article.)

To investigate steaming-induced alterations in lipid metabolites across sweetpotato cultivars, we performed PCA on steamed samples from 10 varieties. PC1 and PC2 cumulatively explained 59.8 % of the total variance (Fig. 5A-B). The PCA Plots of the 10 sweetpotato varieties after steaming showed that P32 could still be clearly separated from the other sweetpotatoes, which was consistent with the raw samples. Compared with the PCA plots of the raw samples, the steamed samples of different sweetpotato varieties were closer together in the PCA plots. We observed the changes in the relative content of lipid metabolites after steaming by constructing a clustered heat map (Fig. 5C). The clustering among varieties corresponded to the PCA plot, and their lipid metabolite accumulation patterns were mainly categorized into three clusters, with the lipid metabolites in cluster 1 mainly having higher accumulation in G79. Lipid metabolites of cluster 2 mainly accumulated more inP32, AN, G87, GZ2 and ZZ3, while lipid metabolites of cluster 3 mainly accumulated more in P32 and G79. The clustered heat maps showed that there were also some differences among different sweetpotatoes after steaming, compared with the clustered heat maps of lipid metabolites of raw samples, indicating that steaming had some effects on lipid metabolites of sweetpotato.

**Supplementary Fig S3:**
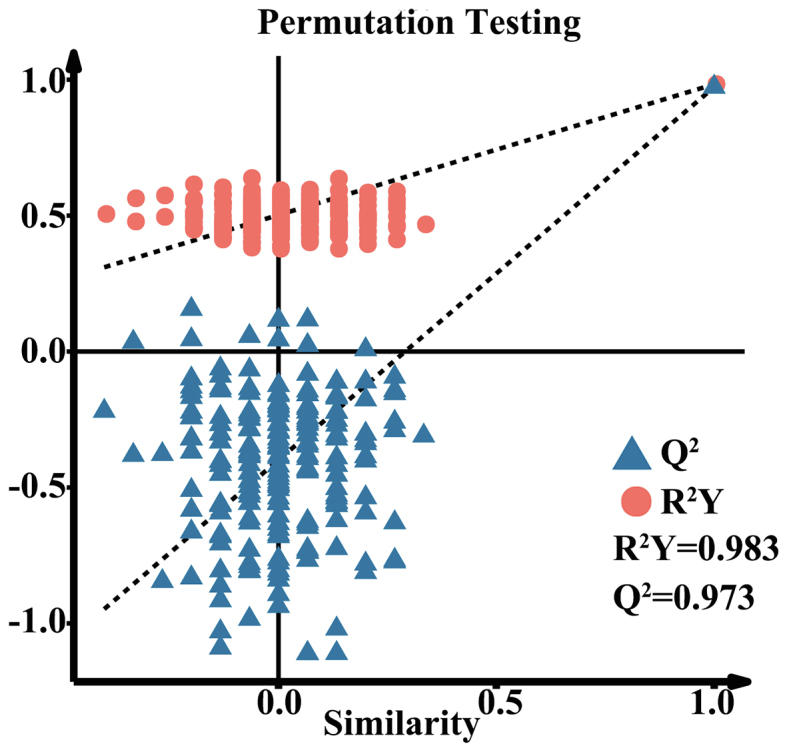

To further investigate the effect of steaming on lipid production in sweetpotato, we first performed PCA analysis on lipid metabolites of 10 sweetpotato varieties before and after steaming, and the results were shown (Fig. 5D). The first principal component (PC1) and second principal component (PC2) explained 46.28 % and 17.07 % of the variance, respectively, totaling 63.35 %. The negative distribution of raw samples in PC1 axis and the positive distribution of steamed samples in PC1 axis indicate that PC1 may be related to temperature. While the PC2 axis mainly separated the different varieties of sweetpotato. PCA results revealed that steaming significantly altered sweetpotato lipid profiles, with distinct differences observed between raw and steamed samples. Therefore, we further used the OPLS-DA model with raw samples as controls to reflect the relationship between sweetpotato lipid metabolites before and after steaming, and plotted score plots (Fig. 5E) and replacement test plots (Fig.S3). The OPLS-DA model had a Q^2^ of 0.973 and an R^2^Y of 0.983, which indicated that the constructed model was usable. The OPLS-DA score plots explained a total of 44.7 % of the variance and the was able to significantly separate raw samples from steamed samples on the PC1 axis. Lipid DAMs were screened based on a combination of VIP values (≥1), *P* < 0.05, and |log2(fold change)| ≥ 1.

**Fig. 6:**
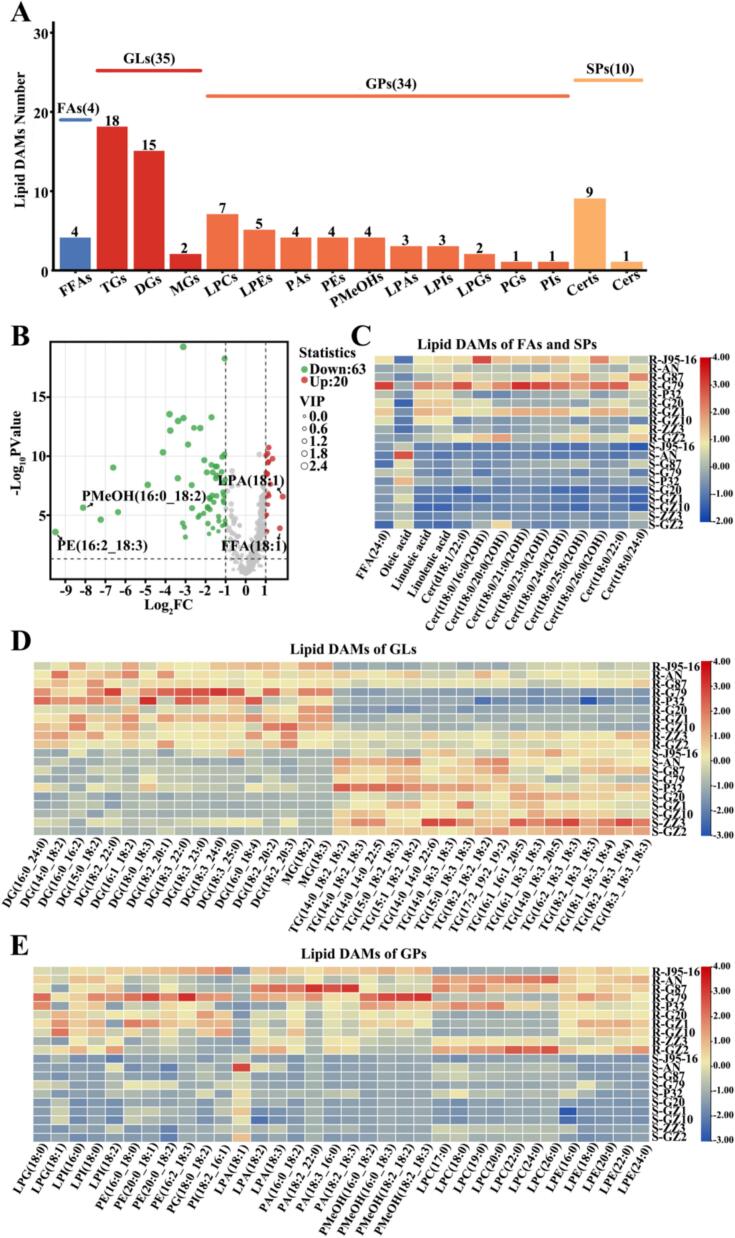
Lipid DAMs analysis of 10 sweetpotato species before and after steaming. (A) Statistics of lipid differential metabolite classes. (B) Volcano plots of lipid DAMs. The red, green, and blue dots indicate the upregulated lipid, the downregulated lipid, and the lipid in which the difference in expression was non-significant, respectively. (C-E) Heatmap visualization of lipid DAMs. Red squares indicate up-regulated metabolites, blue squares indicate down-regulated metabolites, and yellow squares indicate the average relative expression intensity of all metabolites. (For interpretation of the references to colour in this figure legend, the reader is referred to the web version of this article.)

A total of 83 lipid DAMs were identified in the comparison of steamed and raw samples, which were classified into 4 classes and 16 subclasses according to their chemical structures (Fig. 6A, Table S5). The major classes were mainly GPs and GLs, and the subclasses were mainly TGs, DGs, LPCs, LPEs, Certs and FFAs. The volcano plot showed that 63 lipid DAMs were down-regulated and 20 DAMs were up-regulated, suggesting that steaming can lead to changes in lipid metabolites to a certain extent (Fig. 6B). 25 lipid DAMs with VIP > 2 were identified, including oleic acid, linoleic acid, DG (14:0_18:2), DG (16:1_18:2), DG (18:3_24:0), DG (18:2_20:2), MG (18:2), MG (18:3), LPA (18:1), LPE (16:0), and LPE (18,0). These lipids might be key contributors to discriminating raw and steamed sweetpotato, as revealed by VIP analysis. For better visualization, we categorized and plotted heat maps to compare lipid DAMs before and after steaming in different varieties of sweetpotato. In FAs, four lipid DAMs were identified, of which tetracosanoic acid was the only saturated fatty acid and was down-regulated after steaming. Steaming significantly decreased linolenic and linoleic acid levels. These FFAs serve as key precursors for VOCs formation during thermal processing, contributing to the generation of important sweetpotato aromas including benzaldehyde, hexanal, and (E,E)-2,4-decadienal (Li et al., 2021; Liu, Liu, et al., 2023). Whereas oleic acid was up-regulated after steaming, this could be caused by high temperature-induced hydrolysis of TGs to produce FFAs (Barthet et al., 2002). Among the SPs were mainly Certs, all of which were down-regulated after steaming, possibly due to their oxidative degradation at high temperatures. Among the GLs, mainly TGs and DGs, all DGs were down-regulated after steaming, possibly due to thermal degradation or hydrolysis to generate FFAs (Bi et al., 2025). TGs was up-regulated after steaming, which may be due to the decomposition of phosphate groups in PEs during thermal processing, and TGs had good stability during heat treatment (Cao et al., 2025). In GPs, only LPA(18,1) was up-regulated, while the rest were down-regulated. Among them, the main ones were LPCs and LPEs, with small amounts of PEs and PAs. Due to the large number of polyunsaturated bonds, PEs is more prone to oxidation and hydrolysis (Liu, Guo, et al., 2023).

### 3.6 Correlation analysis of sweetpotato VOCs and lipid metabolites after steaming

**Fig. 7:**
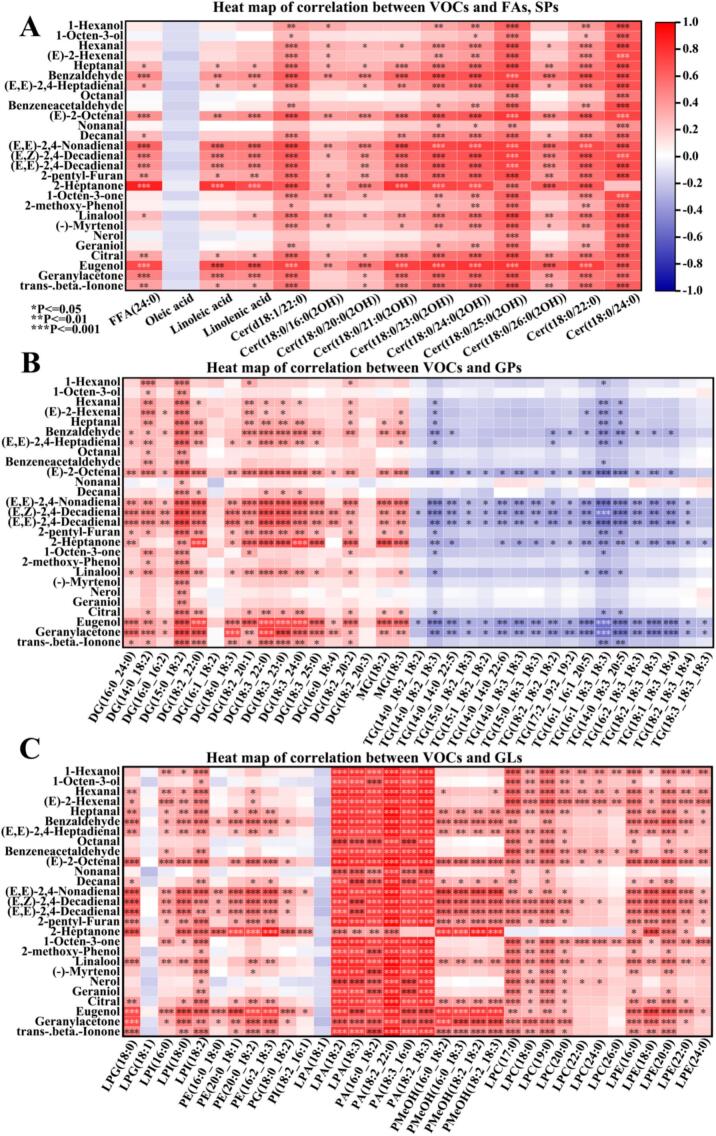
Heat map of the correlation between lipid DAMs and volatile organic compounds. Positive and negative correlations are shown in red and blue, respectively, where *,**,*** indicates significance. (For interpretation of the references to colour in this figure legend, the reader is referred to the web version of this article.)

Fatty acid oxidation can produce VOCs, which suggests that there was a link between lipids and the generation of VOCs (Hu et al., 2023). Based on our previous study of these 10 sweetpotato product VOCs, a total of 27 VOCs with OAV >1 were identified (Jiang et al., 2024; Yao et al., 2024). To further understand the relationship between lipids DAMs and VOCs, we visualized the relationship between VOCs with OAV > 1 and lipid DAMs before and after steaming in 10 sweetpotato varieties through correlation heat map. In this study, we calculated the Pearson correlation between VOCs of OAV > 1 and lipid DAMs, and the results were shown in Fig. 7. Among the FAs, all of them except oleic acid showed significant positive correlation with most of the VOCs, especially benzaldehyde, (E)-2-Octenal, (E,E)-2,4-Nonadienal, (E,Z)-2,4-Decadienal, (E,E)-2,4-Decadienal, (E,E)-2,4-Decadienal, eugenol, geranylacetone. This was due to the ability of linoleic acid and linolenic acid to generate VOCs through degradation and oxidation. In SPs, especially Certs, there were a significant positive correlation with most VOCs (Fig. 7A). DGs and MGs in GLs were significantly positively correlated with most of the VOCs, while TGs were significantly negatively correlated with most of the VOCs (Fig. 7B). TGs and DGs are able to generate DGs and MGs and release FAs through hydrolysis, and their oxidative decomposition is able to form VOCs (Peng et al., 2024; Xu et al., 2025). Almost all of these TGs were significantly negatively correlated with (E)-2-Octenal, (E,E)-2,4-Nonadienal, (E,Z)-2,4-Decadienal, (E,E)-2,4-Decadienal, 2-Heptanone, eugenol, and geranylacetone. In GPs, except for LPG (18:1) and LPA (18:1), all were significantly positively correlated with VOCs (Fig. 7C), especially PAs, LPCs and LPEs. Among them, the degradation of PE can produce LPEs and FAs, thereby further generating VOCs(Liu et al., 2023). The correlation analysis indicated that lipids in sweetpotato were closely related to sweetpotato flavor.

## 4 Conclusion

In this study, lipid metabolites of 10 sweetpotato varieties before and after steaming were identified and analyzed by LC-ESI-MS/MS, and correlation analysis was carried out with previous quality characteristics and lipids. A total of 581 lipid metabolites were detected in sweetpotato, belonging to 6 categories and 28 subclasses. The lipid metabolite contents of the 10 sweetpotato raw samples ranged from 2877 to 5332 μg/g, with the lipid metabolite content of G79 being 1.3–1.8 times higher than that of the other sweetpotatoes. FAs, GPs and GLs were the more abundant lipid metabolites in sweetpotato, while for subclasses, FFAs, PCs, PEs and TGs were more abundant. 10 sweetpotato raw samples were divided into four groups by PCA. The OPLS-DA model was established to screen out a total of 354 DAMs in three comparative combinations, among which 99 lipid DAMs coexisted in the three comparison combinations. P32 had a higher content of TGs compared to the rest of sweetpotatoes, and it is probable that P32 was more resilient to the environment. Group 2 (G79, GZ1, GZ10) and group 3 (J95–16, G20) had higher content of FFA (20:5) compared to P32. Steaming had some effects on lipid metabolites in sweetpotato. The FAs, PRs content of all varieties decreased significantly after steaming, which may be due to the oxidative decomposition of fatty acid-like substances into VOCs. The GLs content increased in all of them, probably due to the release of phospholipids after the cells were destroyed. A total of 83 lipid differential metabolites were screened in raw and vaporized samples, of which the main ones were TG, DG, LPC, and FFA. FFAs (24:0), linoleic acid, and linolenic acid, which are precursors of VOCs, showed a decrease in their contents after steaming. DGs and MGs were both down-regulated after steaming, whereas TGs was up-regulated in all varieties. Most lipids were significantly positively correlated with VOCs, and linoleic acid and linolenic acid were also significantly positively correlated with VOCs, which was caused by their oxidative degradation. Correlation analysis indicates that lipids are involved in certain sweetpotato flavor formation. In conclusion, in this study, lipid profiles of 10 sweetpotatoes tubers before and after steaming were constructed by LC-ESI-MS/MS for the first time, which can help to enrich the understanding of sweetpotato lipids and analyze the differences in lipids among sweetpotato varieties and before and after steaming, and correlate them with VOCs, which can provide a certain theoretical basis for the analysis of the mechanism of sweetpotato breeding and quality formation.

The following are the supplementary data related to this article.

Supplementary material Supplementary material

## CRediT authorship contribution statement

**Xia Jiang:** Writing – original draft, Visualization, Formal analysis, Data curation. **Yanqiang Yao:** Formal analysis, Data curation. **Rong Zhang:** Writing – review & editing, Methodology, Conceptualization. **Yujuan Zhong:** Funding acquisition. **Zhangying Wang:** Writing – review & editing, Methodology, Funding acquisition, Conceptualization.

## Funding

This work was supported by the Food Nutrition and 10.13039/100005622Health Research Center of Guangdong Academy of Agricultural Sciences (XT202226), the Guangdong Modern Agro-industry Technology Research System (2024CXTD07); CARS-10-Sweetpotato, Guangzhou Science and Technology Plan Project (2023B03J1366); Guangdong Provincial Science and Technology Plan Project (2023B1212060038).

## Declaration of competing interest

The authors declare that they have no known competing financial interests or personal relationships that could have appeared to influence the work reported in this paper.

## Data Availability

Data will be made available on request.
